# The CpxR–GlvR regulatory axis of *Edwardsiella piscicida*: a novel virulence regulatory mechanism integrating glycerol metabolism and oxidative stress adaptation

**DOI:** 10.3389/fcimb.2026.1963223

**Published:** 2026-09-25

**Authors:** Xiancheng Jiang, Hanjie Gu, Yan Wang, Yonghua Hu, Hui Guo, Qingjian Fang

**Affiliations:** 1 College of Fisheries, Guangdong Ocean University, Zhanjiang, China; 2 Institute of Tropical Bioscience and Biotechnology & Sanya Research Institute, Chinese Academy of Tropical Agricultural Sciences, Haikou, China; 3 Zhanjiang Experimental Station, Chinese Academy of Tropical Agricultural Sciences, Zhanjiang, China; 4 Hainan Provincial Key Laboratory for Functional Components Research and Utilization of Marine Bio-resources, Haikou, China

**Keywords:** *Edwardsiella piscicida*, glycerol metabolism, stress resistance, transcription factor, virulence

## Abstract

*Edwardsiella piscicida* is a devastating bacterial pathogen in aquaculture, and in-depth elucidation of its pathogenic mechanisms is essential for establishing effective control strategies; the identification of novel regulatory factors can deepen our understanding of bacterial pathogenesis. Our previous work identified the functionally uncharacterized protein ETAE_2516 as a potential target of the response regulator CpxR of the CpxRA two-component system, a key virulence regulator in this pathogen. Bioinformatic analysis and functional prediction indicated that ETAE_2516 is an AraC-family transcription factor that is potentially involved in glycerol metabolism; it was therefore designated GlvR (Glycerol-linked Virulence Regulator). In this study, an in-frame *glvR* deletion mutant (Δ*glvR*) and its complemented strain (Δ*glvR*C) were constructed by gene knockout, and their phenotypes in metabolism, stress resistance, biofilm formation, and pathogenicity were systematically characterized. Deletion of *glvR* did not affect basal bacterial growth but significantly increased sensitivity to oxidative stress and led to intracellular accumulation of reactive oxygen species. The Δ*glvR* mutant exhibited markedly impaired growth in glycerol-supplemented medium, and the expression of glycerol metabolism-associated genes was significantly downregulated, confirming that GlvR positively regulates glycerol metabolism. Loss of GlvR also repressed the expression of *flhC*, the master regulator of the flagellar regulon, thereby impairing bacterial motility. Infection assays using tilapia epithelial (TSE-04) cells, RAW 264.7 macrophages, and a tilapia (*Oreochromis niloticus*) infection model demonstrated that GlvR is essential for bacterial adhesion to host epithelial cells, resistance to host serum killing, intracellular survival within macrophages, and tissue colonization, and all of these defects were restored to wild-type levels in the complemented strain, verifying that they are specifically attributable to the loss of *glvR*. Analysis of the upstream regulatory mechanism further revealed that CpxR binds directly to the *glvR* promoter region and positively activates its transcription. Collectively, these findings demonstrate that the CpxR–GlvR regulatory axis functions as a central hub integrating carbon metabolism, oxidative stress adaptation, and virulence expression, thereby providing a novel perspective for understanding the host adaptation mechanisms of *E. piscicida* and a potential target for anti-virulence strategies against edwardsiellosis.

## 1 Introduction

*Edwardsiella piscicida* is an intracellular pathogen that threatens aquaculture; it can survive within macrophages and cause systemic septicemia, inflicting substantial losses on economically important fish species such as tilapia (*Oreochromis niloticus*) and turbot (*Scophthalmus maximus*) (Cao et al., 2025; Lin et al., 2025). As a facultative intracellular pathogen, its successful colonization depends not only on classical virulence factors but also, critically, on its metabolic adaptation to the restrictive microenvironment of the host (Lanza et al., 2024; Qin et al., 2020). In particular, when bacteria transition from a glucose-rich extracellular environment into the glucose-poor, lipid/glycerol-rich intracellular phagosome, they typically face multiple stresses, including acidification, intensified oxidative stress, and limited nutrient availability, and must therefore reprogram their carbon metabolism—shifting from glycolysis toward glycerol/fatty acid utilization—to sustain energy supply and resist host oxidative killing (Eisenreich et al., 2013; Westman and Grinstein, 2021). However, the transcriptional regulatory mechanisms by which this bacterium senses nutrient shifts in the host environment and coordinates metabolic adaptation with virulence expression remain unclear.

Recent studies have demonstrated that the pathogenicity of intracellular pathogens is tightly coupled to the flexibility of their carbon metabolism (Lee et al., 2013). Pathogens such as *Salmonella* maintain intracellular survival by activating glycerol metabolism pathways to cope with oxidative and nitrosative stresses (Chakraborty et al., 2020; Mao et al., 2022b); these findings indicate that glycerol metabolism is not merely a carbon source utilization pathway but also an important metabolic strategy by which intracellular pathogens adapt to the host environment. Although *Edwardsiella* possesses an intact glycerol utilization gene cluster, the regulatory hub that responds to nutrient fluctuations and the upstream signal-sensing mechanism have not yet been elucidated (Wang et al., 2009). Moreover, existing studies have largely focused on classical virulence apparatuses such as the type III secretion system (T3SS), whereas a systematic understanding of the transcriptional regulatory hierarchy at the metabolism–virulence nexus is still lacking, which limits a complete appreciation of the adaptive survival strategies of intracellular bacteria (Leung et al., 2019).

The physiological activities of Gram-negative bacteria are governed by complex transcriptional regulatory networks, in which AraC-family transcriptional regulators play crucial roles. A hallmark of this protein family is a highly conserved C-terminal DNA-binding domain, which enables these regulators to recognize specific promoter sequences and thereby activate or repress the transcription of downstream target genes (Gallegos et al., 1997; Rodgers and Schleif, 2009). Recent studies have shown that AraC-family transcriptional regulators are extensively involved in environmental responses, carbon metabolism regulation, and virulence gene expression, serving as important transcriptional nodes that connect external signals to downstream physiological programs (Gallegos et al., 1997; Tobes and Ramos, 2002; Yang et al., 2011). For instance, ToxT in *Vibrio cholerae* coordinately activates the expression of cholera toxin and the toxin-coregulated pilus to promote pathogenesis, whereas RarA in *Klebsiella pneumoniae* regulates the expression of the AcrAB efflux pump, thereby enhancing bacterial tolerance to multiple classes of antimicrobial agents (Higgins et al., 1992; Veleba et al., 2012). Upstream two-component systems (such as CpxRA) can regulate such transcription factors to construct hierarchical regulatory networks in response to signals including envelope stress (Nakayama and Watanabe, 1998). Nevertheless, in *E. piscicida*, the specific functions of AraC-family members, the manner in which they mediate the coupling between metabolism and virulence, and whether they are under the direct control of CpxR all remain unknown. This knowledge gap hampers the complete delineation of the transcriptional regulatory network by which this pathogen integrates environmental signals with virulence expression.

In this study, we identified and characterized ETAE_2516, a novel AraC-family transcription factor in *E. piscicida*, and formally designated it GlvR (Glycerol-linked Virulence Regulator) on the basis of its functional characteristics. We elucidated the biological functions of GlvR in *E. piscicida* and defined its role in resistance to oxidative stress and host serum killing; we resolved its positive regulatory effects on flagellar motility, host cell adhesion, and *in vivo* virulence of *E. piscicida*; and we further clarified the relationship between *glvR* and the upstream envelope stress-responsive CpxR regulatory system. These findings provide a theoretical basis for understanding the intracellular adaptation and pathogenic mechanisms of *E. piscicida*.

## 2 Materials and methods

### 2.1 Strains, plasmids, cell lines, and media

The strains, plasmids, and cell lines used in this study are listed in **Supplementary Table 1**. *E. piscicida* was cultured in LB medium at 28 °C, while *Escherichia coli* was cultured in LB medium at 37 °C. TSE-04 (tilapia skin epithelial) cells were maintained in L-15 medium (Biosharp, Hefei, China) supplemented with 10% fetal bovine serum (FBS; Gibco, USA) at 28 °C. RAW 264.7 cells were maintained in high-glucose DMEM (Solarbio, Beijing, China) supplemented with 10% FBS at 37 °C in a 5% CO_2_ atmosphere. Antibiotics were used at the following final concentrations: polymyxin B (poly B, Biodee, Beijing, China), 20 μg/mL; chloramphenicol (Cm, Yuanye, Shanghai, China), 20 μg/mL; and ampicillin (Amp, Biosharp, Hefei, China), 50 μg/mL.

### 2.2 Bioinformatics analysis of GlvR

The amino acid sequence of the *E. piscicida* GlvR protein was retrieved from NCBI (https://www.ncbi.nlm.nih.gov/), and sequence alignment was performed using Jalview 2.11.3.2 and ESPript 3.0 (https://espript.ibcp.fr/ESPript/cgi-bin/ESPript.cgi). Topology annotation, functional domain prediction, and three-dimensional structure analysis of the amino acid sequence were performed using the online tools Protter (http://wlab.ethz.ch/protter/start/), SMART (http://smart.embl-heidelberg.de/), and SWISS-MODEL (https://swissmodel.expasy.org/), respectively.

### 2.3 Construction of the deletion mutant and the complemented strain

To construct the *glvR* deletion mutant, the upstream and downstream flanking fragments of *glvR* were amplified by PCR from *E. piscicida* genomic DNA using the primer pairs GlvKOF1/R1 and GlvKOF2/R2, respectively, and the two fragments were then fused by overlap extension PCR with the primers GlvKOF1/R2. The fused fragment was cloned into the BglII site of the suicide plasmid pDM4, yielding the recombinant plasmid pDM*glvR*. The recombinant plasmid pDM*glvR* was transformed into *E. coli* S17-1λpir, and the resulting strain was mated with the wild-type strain to allow allelic exchange by homologous recombination; the *glvR* deletion mutant was selected on LB plates containing 10% sucrose. The mutant was confirmed by PCR with the primers GlvKOF1/R2 and by 16S rRNA sequencing, and was designated Δ*glvR*. To construct the complemented strain, the *glvR*C fragment was amplified from *E. piscicida* genomic DNA with the primers GlvCF/CR and cloned into the pT3 plasmid (a pBR322 derivative carrying the Ptrc promoter; Zhang et al., 2008) as the ligation vector, yielding the recombinant plasmid pT*glvR*, which was subsequently introduced into Δ*glvR* to generate the complemented strain Δ*glvR*C. Details of all primers used are provided in **Supplementary Table S2**.

### 2.4 Growth curve determination

Bacteria were cultured to the logarithmic growth phase and then diluted 1,000-fold with LB medium. A 200-μL aliquot of each dilution was transferred into a Bioscreen C 96-well honeycomb plate (Bioscreen, Finland), and bacterial growth was monitored using the Bioscreen C automated growth curve analysis system. Each sample was assayed in triplicate, and the experiment was repeated three times.

### 2.5 Transcriptome sequencing and analysis

The WT and Δ*glvR* strains were cultured in LB medium to the logarithmic phase, and three biological replicates were prepared for each strain. Total RNA was extracted using TRIzol reagent (Invitrogen, Carlsbad, USA) according to the manufacturer’s instructions, and RNA concentration, purity, and integrity were assessed with a NanoDrop spectrophotometer and agarose gel electrophoresis. Sequencing libraries were then constructed and sequenced on the Illumina platform. Clean reads were obtained by removing adaptor sequences and low-quality reads from the raw data and were mapped to the *E. piscicida* reference genome. Gene expression levels were quantified, and differentially expressed genes (DEGs) between the WT and Δ*glvR* strains were identified using DESeq2, with *P* < 0.05 and |log2 (fold change)| > 0.585 as the screening criteria. KEGG and GO enrichment analyses of the DEGs were performed using ClusterProfiler, and terms with *P* < 0.05 were considered significantly enriched.

### 2.6 Reverse transcription quantitative PCR analysis

RT-qPCR was performed according to the method of Fang et al. (2023). Briefly, the wild-type (WT) strain, Δ*glvR*, Δ*glvR*C, Δ*cpxR*, Δ*cpxA*, and Δ*cpxRA* strains were cultured in LB medium to the logarithmic phase and collected; in parallel, the WT strain was cultured to the logarithmic phase in basal LB medium or in LB medium supplemented with 30 mM glycerol (Xilong, Guangdong, China) or 30 mM glucose (Solarbio, Beijing, China), and the WT strain was treated with 1 mM H_2_O_2_ (Xilong, Guangdong, China) for 1 h (the control group was treated with PBS). Total RNA was extracted from the bacteria of each group and reverse-transcribed into cDNA. RT-qPCR was performed using cDNA as the template and 16S rRNA as the internal reference gene; the primers used are listed in **Supplementary Table 2**. The relative expression levels of the target genes were calculated using the 2^−ΔΔCt^ method. Each sample was analyzed with three technical replicates, and the experiment was independently repeated three times.

### 2.7 Oxidative stress resistance assay and intracellular reactive oxygen species detection

Bacteria cultured to the logarithmic phase were serially diluted with fresh LB medium and spot-inoculated onto LB plates with or without 0.5 mM H_2_O_2_, followed by incubation at 28 °C for 24 h. Alternatively, logarithmic-phase bacteria were diluted 10-fold with PBS and treated with 1 mM H_2_O_2_ for 1 h (the control group was treated with PBS); after appropriate dilution, the suspensions were spread onto LB plates for colony counting, and the experiment was repeated three times. For the detection of intracellular reactive oxygen species (ROS), the WT, Δ*glvR*, and Δ*glvR*C strains cultured to the logarithmic phase were washed with PBS and resuspended; the cells were lysed by ultrasonication on ice, and the supernatant was collected by centrifugation. The DCFH-DA (Beyotime, Jiangsu, China) fluorescent probe was added to a final concentration of 10 μM, followed by incubation at 28 °C in the dark for 30 min. Fluorescence intensity was measured using a multifunctional microplate reader (excitation wavelength, 488 nm; emission wavelength, 525 nm), and intracellular ROS levels were expressed as relative fluorescence intensity. The experiment was repeated three times.

### 2.8 Biofilm formation and swimming motility assays

The biofilm-forming ability of the bacteria was determined by crystal violet staining, following the procedure described by Fang et al. (2023). For the swimming motility assay, bacteria were cultured to the logarithmic phase, and 1 μL of each culture was spot-inoculated onto LB plates containing 0.3% agar, followed by incubation at 28 °C for 24 h. Bacterial swimming motility was observed, and the diameter of the motility zone was measured. Each sample was assayed in triplicate, and the experiment was repeated three times.

### 2.9 Infection of host cells

TSE-04 cells were cultured in 96-well plates until a confluent adherent monolayer was formed, and 100 μL of bacterial suspension (5 × 10^6^ CFU) was mixed with the TSE-04 cells at a multiplicity of infection (MOI) of 50:1. After incubation at 28 °C for 1 h or 2 h, the cells were washed three times with PBS. To determine the number of bacteria, the cells were lysed with 200 μL of 1% (v/v) Triton X-100 (Solarbio, Beijing, China) for 15 min, and the lysates were appropriately diluted and spread onto LB agar plates for counting; the experiment was repeated three times. To visualize bacterial adhesion to TSE-04 cells more directly, GFP-tagged WT and Δ*glvR* strains were used to infect TSE-04 cells. After 2 h of infection, the cells were fixed with 4% paraformaldehyde (Biosharp, Hefei, China) at room temperature and stained with the cytoskeletal dye phalloidin (Biosharp, Hefei, China) and the nuclear dye DAPI (Roche, Switzerland), respectively. Bacterial adhesion to the cells was observed and photographed under a laser scanning confocal microscope.

The intracellular survival and proliferation of *E. piscicida* in murine macrophages (RAW 264.7) were evaluated according to the method described by Wang et al. (2019). RAW 264.7 cells were cultured in DMEM supplemented with 10% FBS at 37 °C in a 5% CO_2_ incubator until the cells were adherent. A 100-μL aliquot of bacterial suspension (1 × 10^6^ CFU) was added to the cells at an MOI of 10:1. After infection at 28 °C for 2 h, the cells were washed three times with PBS; fresh DMEM containing 200 μg/mL gentamicin was then added, and the cells were incubated at 28 °C in a 5% CO_2_ incubator for 2 h to kill extracellular bacteria. The cells were washed three more times with PBS, fresh DMEM was added, and samples were collected at 0, 2, 4, and 6 h of incubation to determine the number of intracellular bacteria in the RAW 264.7 cells. The experiment was repeated three times.

To visualize bacterial survival and proliferation within RAW 264.7 cells more directly, GFP-tagged WT and Δ*glvR* strains were used to infect RAW 264.7 cells. After 2 h of infection, extracellular bacteria were killed with DMEM containing 200 μg/mL gentamicin, and the medium was replaced with fresh DMEM for further incubation for 0 h and 4 h. The cells were fixed with 4% paraformaldehyde and stained with the nuclear dye DAPI, and bacterial survival was observed and photographed under a laser scanning confocal microscope.

### 2.10 Resistance to host serum

The serum resistance assay was performed mainly according to the method of Hu et al. (2016). Briefly, bacteria were cultured to the logarithmic phase, washed with PBS, and resuspended in PBS. A 50-μL aliquot of bacterial suspension (approximately 10^5^ CFU) was mixed with 50 μL of tilapia serum and incubated at 28 °C for 1 h; the control group was incubated with 50 μL of PBS instead. After appropriate dilution, the mixtures were spread onto plates for colony counting. The experiment was repeated three times. In preliminary experiments, the viability of *E. piscicida* incubated with heat-inactivated tilapia serum (56 °C for 30 min) did not differ significantly from that of bacteria incubated with PBS (Hu et al., 2016); therefore, PBS was used as the control in this assay.

### 2.11 Tilapia infection experiment

Healthy Nile tilapia (*Oreochromis niloticus*; body weight, 10–15 g) were purchased from a commercial tilapia farm in Wenchang, Hainan Province, China, and were acclimated in aerated freshwater at 28 °C for two weeks before the experiments. Bacteria cultured to the logarithmic phase were collected by centrifugation and washed three times with PBS to remove residual LB medium; the bacteria were then resuspended in PBS and diluted to 1 × 10^7^ CFU/mL. Tilapia were intramuscularly injected with 20 μL of the bacterial suspension (30 fish per group), and the control group was injected with an equal volume of PBS. The fish were observed continuously for 14 days, and mortality was recorded. For the determination of tissue bacterial loads, bacterial suspensions were prepared as described above and intramuscularly injected into tilapia (10 fish per group). At 24 h and 48 h post-infection, tilapia were randomly sampled and dissected; the spleen and head kidney were collected under aseptic conditions, weighed, and homogenized in an appropriate volume of sterile PBS. The homogenates were serially diluted, spread onto LB plates, and incubated at 28 °C for 24 h, followed by colony counting to calculate the bacterial load per gram of tissue (CFU/g). The experiment was repeated three times.

### 2.12 Promoter reporter assay and electrophoretic mobility shift assay

The promoter region fragment of *glvR* was amplified with the primers GlvR-PF/R and cloned into the pSC11 plasmid, yielding the promoter reporter plasmid pS*glvR*. The *cpxR* fragment was cloned into the pT3 plasmid, yielding the recombinant plasmid pT*cpxR*. The pT3 and pT*cpxR* plasmids were transformed into *E. coli* DH5α to obtain DH5α/pT3 and DH5α/pT*cpxR*, respectively, and pS*glvR* was then transformed into DH5α/pT3 and DH5α/pT*cpxR* to obtain the promoter reporter strains DH5α/pS*glvR*/pT3 and DH5α/pS*glvR*/pT*cpxR*. Following the same procedure, the promoter region fragments of *glpD*, *glpT*, and *glpA* were amplified and cloned into the pSC11 plasmid to generate the promoter reporter plasmids pS*glpD*, pS*glpT*, and pS*glpA*, respectively, and the *glvR* fragment was cloned into the pT3 plasmid to generate the recombinant plasmid pT*glvR*. The pS*glpD*, pS*glpT*, and pS*glpA* plasmids were co-transformed with either pT3 or pT*glvR* into DH5α to obtain the corresponding promoter reporter strains. Details of all primers used are provided in **Supplementary Table 2**. β-Galactosidase activity was measured according to the method of Ma et al. (2021). The experiment was repeated three times.

The electrophoretic mobility shift assay (EMSA) was performed according to the method of Fang et al. (2023). The *glvR* promoter region fragment was incubated with the CpxR protein or the TrxA protein (control protein), and the incubation products were subjected to non-denaturing PAGE. After staining with a green fluorescent dye, the gels were visualized and photographed using a gel imaging system (Tanon, Shanghai, China). The experiment was repeated three times.

### 2.13 Statistical analysis

Data analysis was performed using SPSS 25.0 software (IBM, Armonk, USA). The data were first examined for obvious outliers and tested for normality. Comparisons between two groups were performed using the independent-samples *t*-test; comparisons among multiple groups were performed using one-way analysis of variance (one-way ANOVA), followed by the Tukey–Kramer test for multiple comparisons. Differences in tilapia survival rates among groups were analyzed using the log-rank test. Data are presented as the mean ± standard error of the mean (SEM). Statistical significance was set at *P* < 0.05 (*), and high statistical significance at *P* < 0.01 (**).

## 3 Results

### 3.1 Bioinformatic analysis of GlvR in *Edwardsiella piscicida*

AraC-family transcription factors are key nodes connecting environmental signals to bacterial physiological responses; however, in *E. piscicida*, some AraC-family members remain functionally uncharacterized beyond the known regulators. Protein BLAST and sequence alignment analyses revealed that GlvR is highly conserved across *E. piscicida*, *Edwardsiella ictaluri, Obesumbacterium proteus, and Hafnia alvei*, sharing 94.26%, 71.31%, and 70.49% sequence similarity with its homologs in the latter three species, respectively, and all these homologs contain a conserved C-terminal helix-turn-helix (HTH) signature motif (**Figure 1A**). Analysis of protein sequence features, including signal peptide and transmembrane topology prediction, showed that the protein encoded by ETAE_2516 contains neither a signal peptide nor transmembrane helices, indicating that it is an intracellularly localized protein (**Figure 1B**). Functional domain and three-dimensional structure analyses revealed a DNA-binding domain at the C terminus of the protein (amino acids 31–110) (**Figure 1C**). Homology modeling and molecular docking analyses further confirmed (**Figure 1D**) that the C terminus of this protein contains a typical AraC-family DNA-binding domain, which is composed of seven α-helices forming a canonical HTH (helix-turn-helix) motif. The structural model showed that one of these helices, the recognition helix, can specifically insert into the major groove of DNA to achieve specific DNA binding. Molecular docking analysis further predicted that the residues ILE28, TYR37, ALA55, ALA56, and GLY105, which are located within or adjacent to the HTH motif and are highlighted in magenta in **Figure 1D**, constitute the key amino acids mediating the interaction between GlvR and DNA.

**Figure 1:**
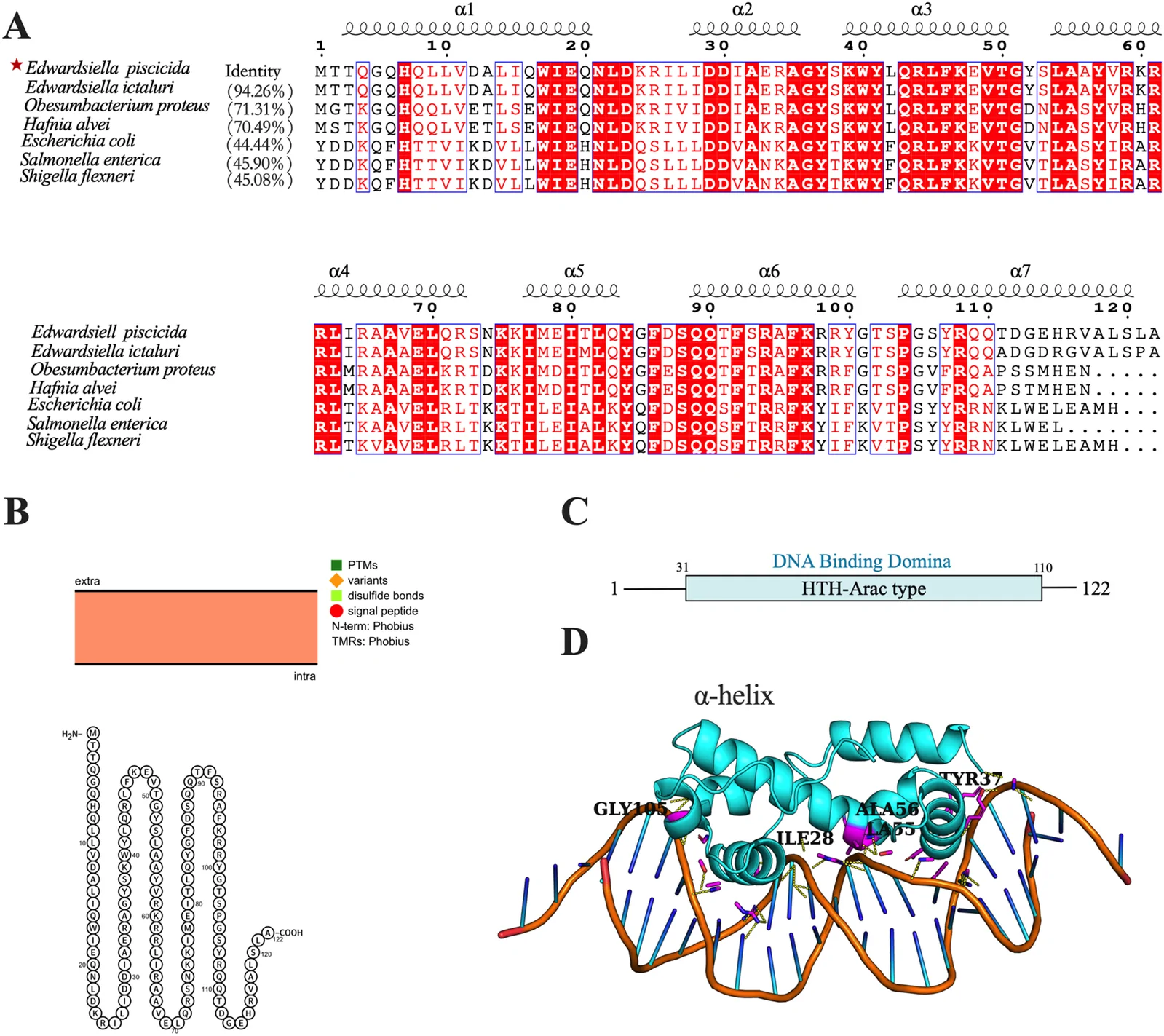
Bioinformatic analysis of the GlvR protein of *E. piscicida*. **(A)** Amino acid sequence alignment of GlvR from *E. piscicida* and its homologs. Residues with 100% sequence identity are shown as white text on a red background, and residues with 75% identity are shown as red text on a white background. **(B)** Schematic diagram of the sequence feature analysis. **(C)** Schematic diagram of the protein functional domains. The AraC-family helix-turn-helix (HTH-AraC type) DNA-binding domain (residues 31–110) is annotated. **(D)** Homology modeling and molecular docking analysis. Key amino acid residues predicted to be involved in DNA binding (GLY105, ILE28, ALA56, TYR37, and ALA55) are highlighted in magenta.

### 3.2 Construction of the *glvR* deletion and complemented strains and transcriptome analysis of GlvR

To investigate the biological role of GlvR in *E. piscicida*, the in-frame 79–312 bp fragment of *glvR* was deleted in this study. PCR and sequencing verification confirmed the successful in-frame deletion of *glvR*, yielding the deletion mutant Δ*glvR* (**Figure 2A**). Using the pT3 plasmid as the complementation vector, the recombinant plasmid pT*glvR*C was introduced into the mutant Δ*glvR*, and the complemented strain Δ*glvR*C was successfully constructed (**Figure 2B**).

**Figure 2:**
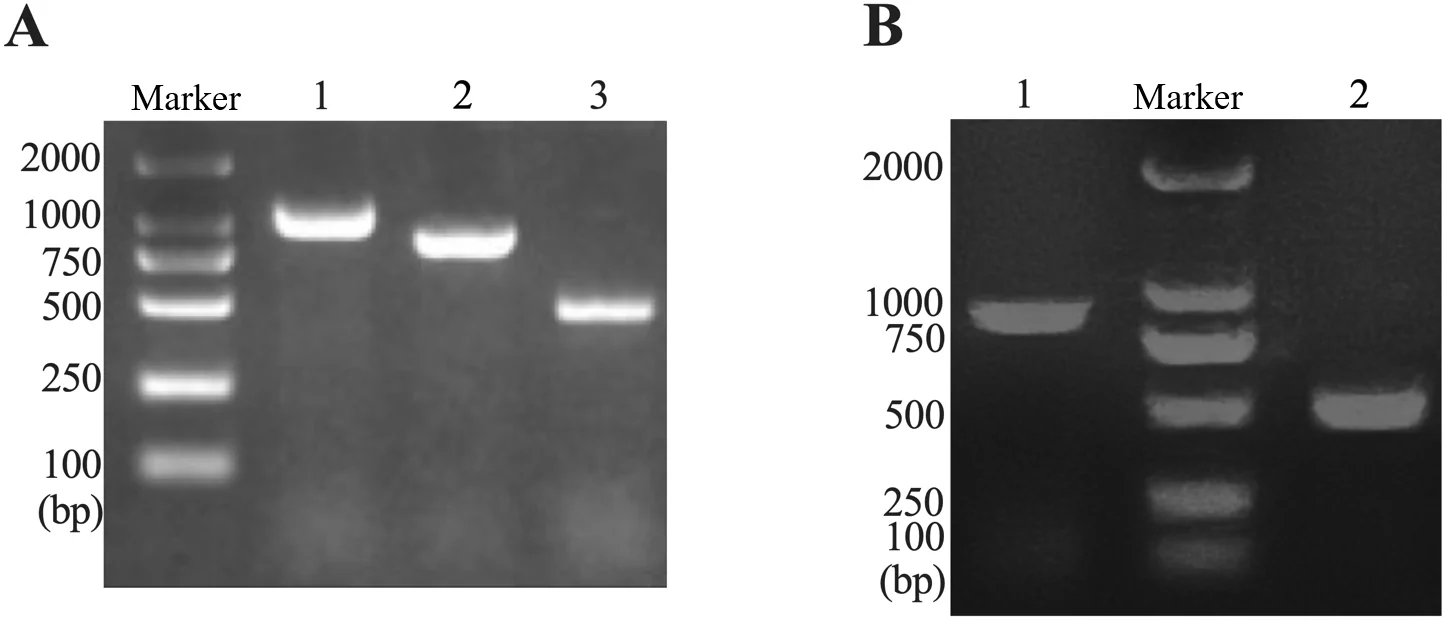
Construction of the *glvR* deletion and complemented strains. **(A)** PCR verification of the *E. piscicida glvR* deletion strain. Lane 1, wild-type strain; lane 2, *glvR* deletion mutant; lane 3, detection with *E. piscicida*-specific primers. **(B)** PCR verification of the *E. piscicida glvR* complemented strain. Lane 1, wild-type strain; lane 2, detection with *E. piscicida*-specific primers.

To further delineate the downstream regulon of GlvR and to screen for key functional pathways in which it may be involved, transcriptome sequencing analysis was performed on the wild-type and Δ*glvR* deletion strains. Using *P* < 0.05 and |log2 (fold change)| > 0.585 (fold change > 1.5) as the screening criteria, a total of 108 significantly differentially expressed genes (DEGs) were identified, of which 50 were upregulated and 58 were downregulated (**Figure 3A**). Compared with the WT, multiple genes associated with glycerol uptake, transport, and metabolism were significantly downregulated in Δ*glvR*, including *glpD*, *glpA*, *glpT*, *glpQ*, *glpF*, and *glpK*. In addition to these genes, several genes related to redox and energy metabolism also exhibited altered expression, such as *trxA*, *ssaT*, *dcuA*, ETAE_RS11625, and ETAE_RS04375. These results indicated that the genes affected by *glvR* deletion were mainly concentrated in glycerol metabolism and related physiological processes. To further resolve the functional distribution of the DEGs, KEGG and GO enrichment analyses were performed. The KEGG enrichment results showed that the DEGs were mainly involved in pathways such as glycerophospholipid metabolism, bacterial invasion of epithelial cells, and *Yersinia* infection (**Figure 3B**). The GO enrichment results indicated that these genes were mainly enriched in processes such as glycerol transmembrane transport, glycerol-3-phosphate metabolism, and pyrimidine nucleotide salvage (**Figure 3C**). Taken together, the transcriptional changes caused by GlvR deletion exhibited a clear functional focus, mainly concentrating on glycerol uptake, transport, and related downstream metabolic processes.

**Figure 3:**
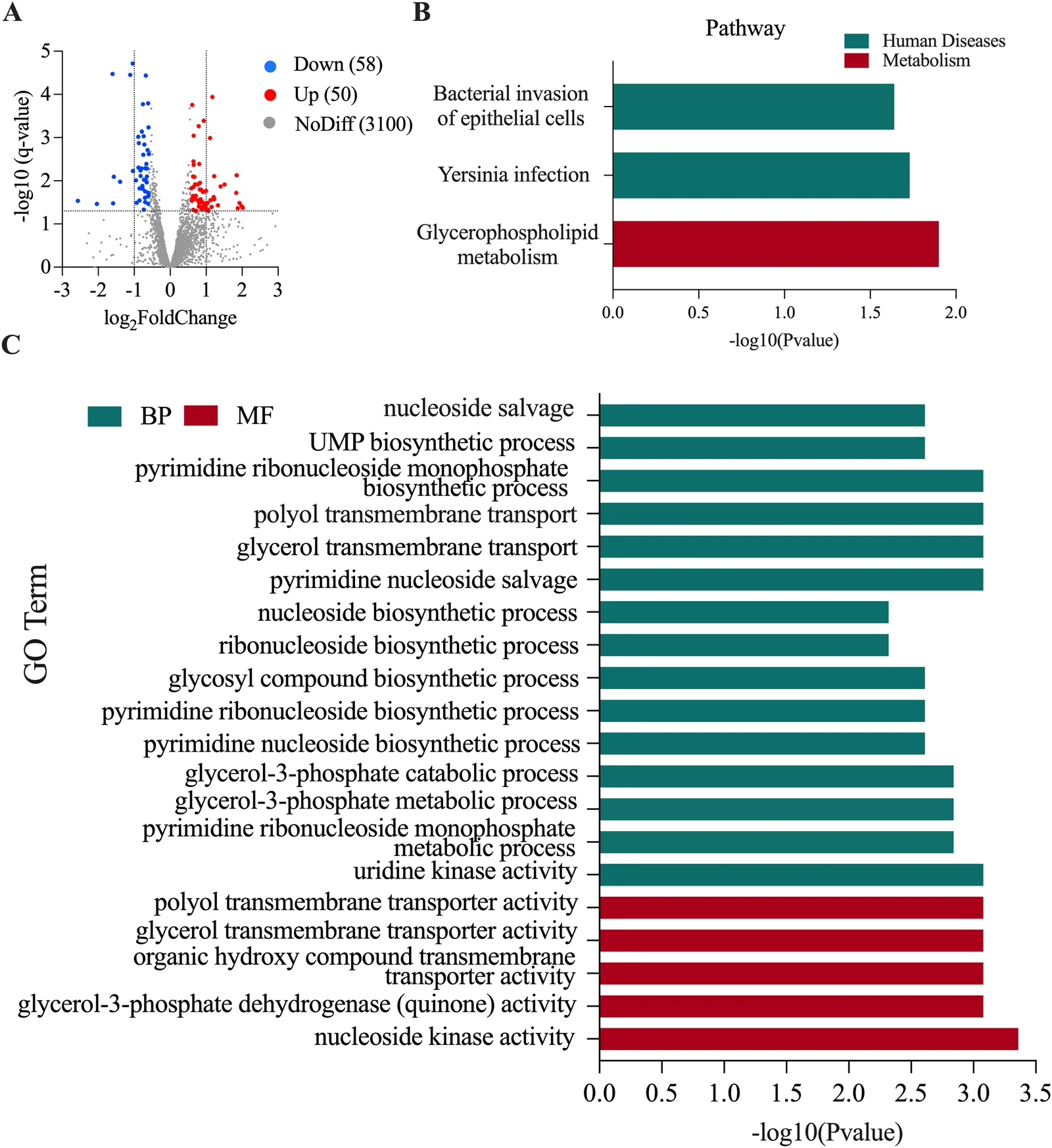
Differentially expressed genes (DEGs) and KEGG/GO enrichment analysis of the *glvR* deletion strain. **(A)** Volcano plot of the DEGs. **(B)** KEGG pathway classification and statistics of the DEGs. Enrichment analysis was performed using ClusterProfiler, and P-values were calculated by the hypergeometric distribution method (*P*-value < 0.05). **(C)** Analysis of significantly enriched GO terms among the DEGs; the analysis method was the same as described above.

### 3.3 GlvR is involved in glycerol metabolism of *E. piscicida*

Given that the transcriptome analysis suggested that *glvR* may play an important regulatory role in glycerol utilization and related metabolic remodeling in *E. piscicida*, we further investigated its function in glycerol metabolism. First, a subset of the DEGs was validated by RT-qPCR, and the results were highly consistent with the RNA-seq trends (**Figure 4A**). Subsequently, the transcription levels of *glvR* in *E. piscicida* cultured in basal LB medium, LB medium supplemented with glycerol, and LB medium supplemented with glucose were measured. The results showed that, compared with basal LB medium or LB medium supplemented with glucose, glycerol supplementation significantly upregulated the transcription of *glvR* (**Figure 4B**), indicating that *glvR* expression is induced by glycerol. Further growth analysis revealed no significant differences in growth among the strains in basal LB medium (**Figure 4C**); however, in LB medium supplemented with glycerol, the endpoint growth (OD_600_) of Δ*glvR* after 12 h of incubation was significantly lower than that of the WT and Δ*glvR*C (**Figure 4D**). To further verify the regulatory effect of GlvR on glycerol metabolism-related genes, three representative genes that were significantly downregulated in the transcriptome, *glpD*, *glpT*, and *glpA*, were selected for promoter reporter assays. These three genes were chosen because they were among the most significantly downregulated glycerol metabolism-related DEGs and collectively represent the key steps of glycerol uptake and catabolism: *glpT* encodes the glycerol-3-phosphate transporter responsible for substrate uptake, whereas *glpD* and *glpA* encode the aerobic and anaerobic glycerol-3-phosphate dehydrogenases, respectively, which catalyze the central catabolic step of glycerol utilization. The results showed that, compared with the empty-vector control, expression of GlvR significantly increased the β-galactosidase activity driven by the *glpD*, *glpT*, and *glpA* promoters (**Figures 4E–G**), indicating that GlvR positively regulates the transcription of these three glycerol metabolism-related genes. Taken together, these results demonstrated that GlvR promotes glycerol utilization in *E. piscicida* by positively regulating multiple glycerol metabolism-related genes.

**Figure 4:**
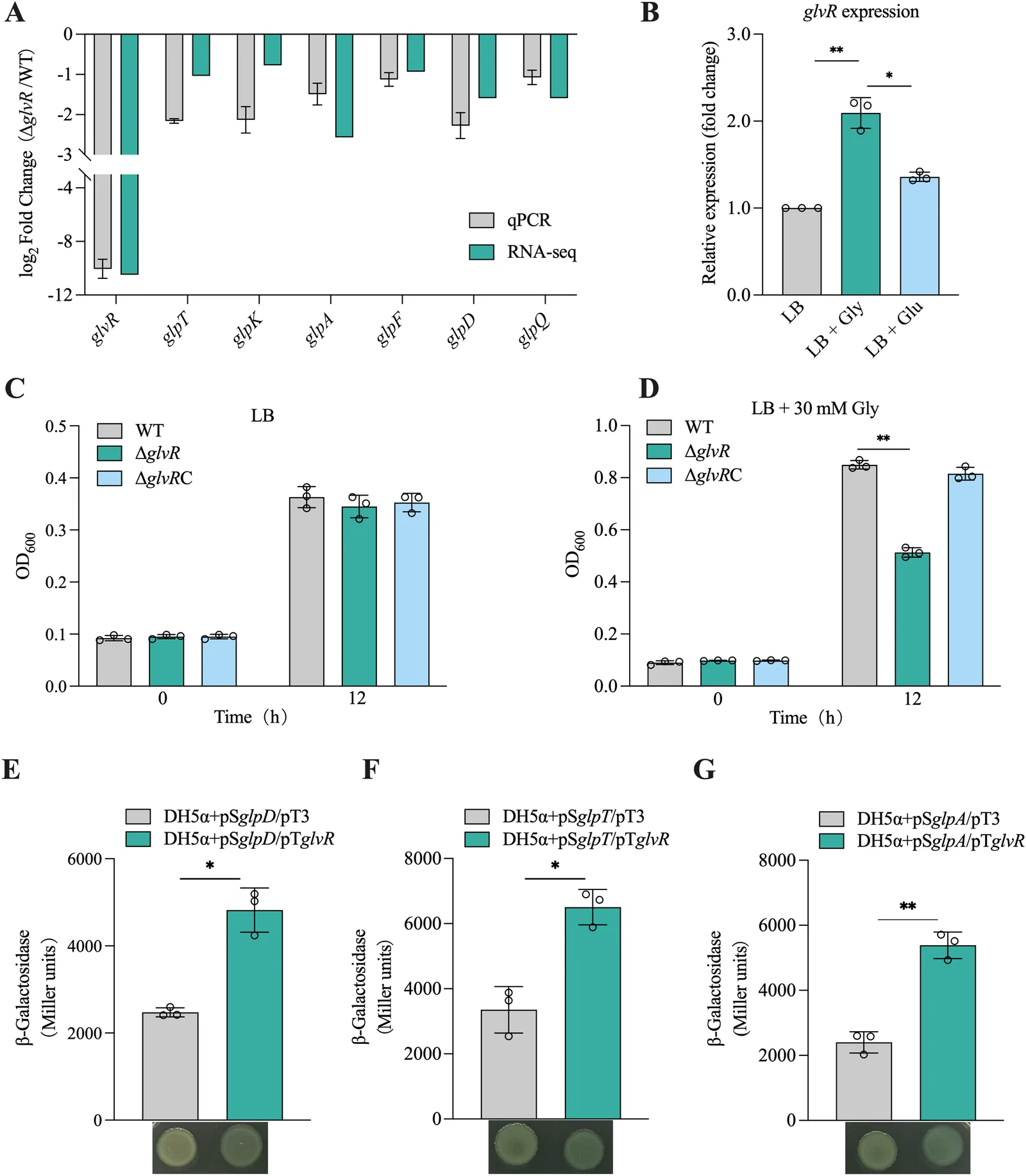
Involvement of *glvR* in glycerol metabolism of *E. piscicida*. **(A)** Validation of the RNA-seq data by RT-qPCR. **(B)** Transcription levels of *glvR* in LB medium supplemented with glycerol (Gly) or glucose (Glu), as detected by RT-qPCR; the transcription level in standard LB medium served as the control. **(C)** Growth of the strains in LB medium. OD_600_ was measured for the WT, Δ*glvR*, and Δ*glvR*C strains after 0 h and 12 h of incubation in standard LB medium. **(D)** Growth of the same strains in LB medium supplemented with 30 mM glycerol; OD_600_ was measured at 0 h and 12 h. **(E–G)** β-Galactosidase activities driven by the promoters of *glpD* **(E)**, *glpT* **(F)**, and *glpA* **(G)** in the presence of GlvR overexpression or an empty-vector control. Data are presented as the mean ± SEM of three independent experiments; two-group comparisons **(A, E–G)** were analyzed using the independent-samples t-test, and multi-group comparisons **(B–D)** were analyzed using one-way ANOVA followed by the Tukey–Kramer test; *P < 0.05, **P < 0.01.

### 3.4 Role of GlvR in the stress resistance of *E. piscicida*

Glycerol metabolism not only participates in carbon source uptake and energy supply; its key intermediate, glycerol-3-phosphate, is also an important precursor of bacterial glycerophospholipid biosynthesis and is closely associated with membrane lipid homeostasis and redox balance (Dowhan and Bogdanov, 2021; Parsons and Rock, 2013). Previous studies have shown that the remodeling of bacterial lipid metabolism is an important adaptive strategy for coping with stressful environments, and that disruption of glycerol-3-phosphate homeostasis can further affect bacterial growth, physiological adaptation, and virulence-related processes (Liu et al., 2022). On this basis, we hypothesized that GlvR-mediated regulation of glycerol metabolism may further participate in the stress responses of *E. piscicida*; therefore, the viability of the strains under normal conditions and under hydrogen peroxide stimulation was further analyzed. First, the growth phenotypes of the WT, Δ*glvR*, and Δ*glvR*C strains under hydrogen peroxide stress were examined. Under normal conditions, the growth and tolerance of Δ*glvR* did not differ significantly from those of the WT (**Figure 5A**), whereas under hydrogen peroxide stress, the growth of Δ*glvR* was markedly weaker than that of the WT strain, and the phenotype of the complemented strain Δ*glvR*C was largely restored (**Figure 5B**). Growth curves were then determined in the presence of 0.5 mM H_2_O_2_, and survival rates were measured after treatment with 1 mM H_2_O_2_. The results showed that the growth of the Δ*glvR* strain was significantly inhibited under oxidative stress, and its survival rate (38%) was significantly lower than that of the wild-type strain (76%), indicating a markedly increased sensitivity to oxidative stress (**Figures 5B, C**); moreover, the intracellular ROS level of Δ*glvR* was significantly higher than that of the WT (**Figure 5D**). Further analysis revealed that, upon H_2_O_2_ stimulation, the expression levels of glycerol metabolism-related genes in the WT strain were significantly upregulated compared with the untreated group (**Figure 5E**), suggesting that glycerol metabolism-related processes in *E. piscicida* are closely associated with the oxidative stress response. In summary, GlvR plays an important role in the stress responses of *E. piscicida*, particularly in its tolerance to oxidative stress.

**Figure 5:**
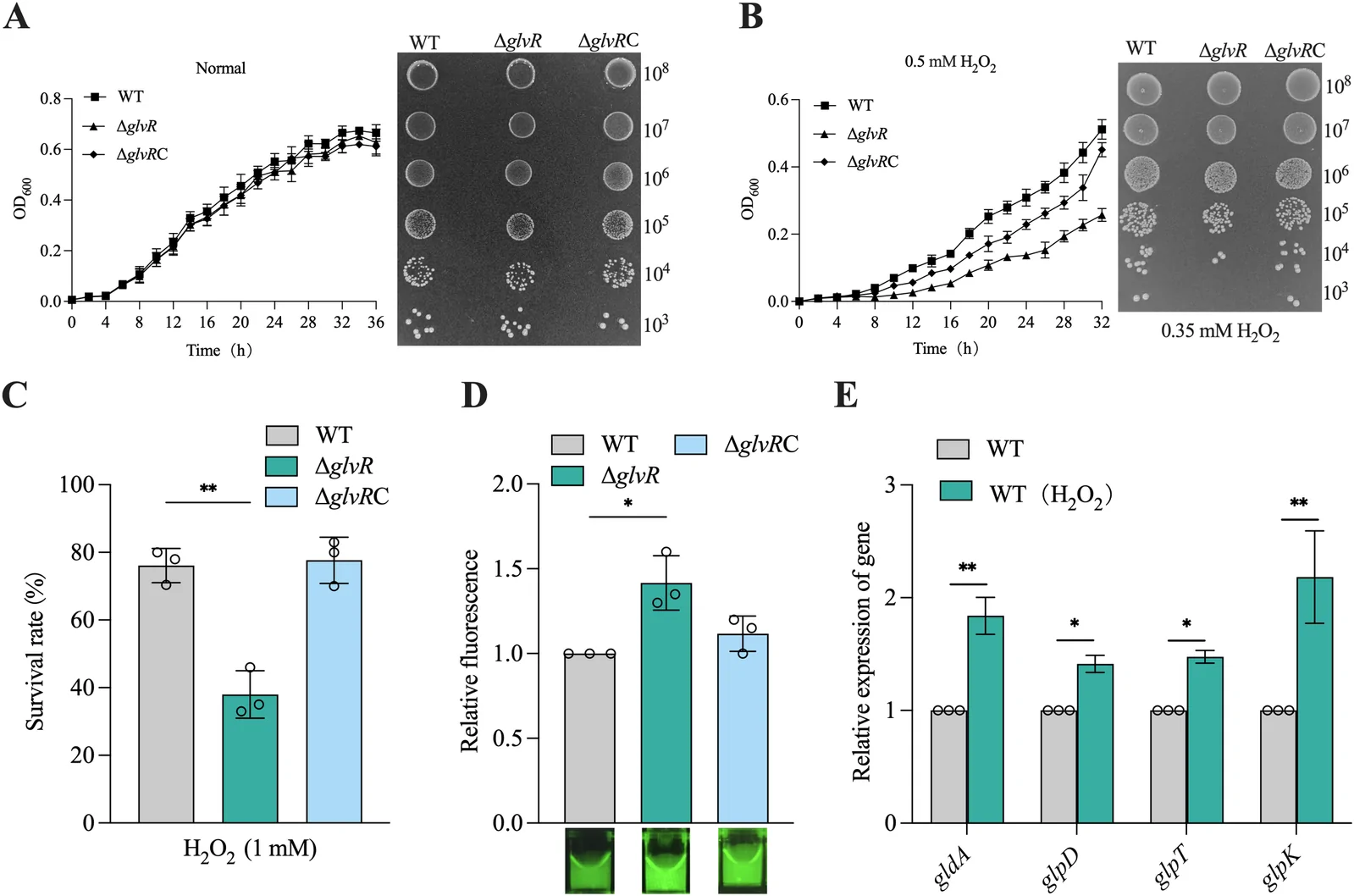
Effect of *glvR* on the stress resistance of *E. piscicida*. **(A)** Effect of *glvR* deletion on bacterial growth. Left panel: growth curves of the wild-type, Δ*glvR* mutant, and complemented strains under normal conditions; right panel: growth of serially diluted bacterial cultures on agar plates. **(B)** Growth in LB medium under oxidative stress induced by 0.5 mM H_2_O_2_. **(C)** Determination of the survival rates of *E. piscicida* under 1 mM H_2_O_2_ stress. **(D)** Detection of intracellular ROS levels. The WT, Δ*glvR*, and Δ*glvR*C strains were incubated with the DCFH-DA fluorescent probe at 28 °C with shaking for 30 min, and the relative intracellular ROS levels were measured. **(E)** Transcription levels of glycerol metabolism-related genes after H_2_O_2_ stimulation, as detected by RT-qPCR. All quantitative data are presented as the mean ± SEM of three independent experiments; and two-group comparisons were analyzed using the independent-samples t-test; *P < 0.05, **P < 0.01.

### 3.5 Effects of GlvR on the motility and biofilm formation of *E. piscicida*

Bacterial motility and biofilm-forming capacity are considered important phenotypic factors that promote host colonization and enhance pathogenicity (Shi et al., 2019; Xu et al., 2014). To investigate the biological role of GlvR in *E. piscicida*, the effect of *glvR* deletion on basal bacterial motility was first evaluated. Motility assays of the WT, Δ*glvR*, and Δ*glvR*C strains were performed on LB semi-solid plates containing 0.3% agar. The results showed that the motility diameter of the Δ*glvR* strain was significantly reduced compared with those of the WT and Δ*glvR*C (**Figure 6A**), indicating that *glvR* deletion impaired the motility of *E. piscicida*. The transcription levels of the flagellar regulatory gene *flhC* and its downstream genes *fliA* and *fliC* were further examined, and the results showed that the expression of all of these genes was significantly decreased in Δ*glvR* (**Figure 6B**), suggesting that *glvR* may participate in the regulation of bacterial motility by controlling the expression of flagellum-related genes. In addition, to investigate whether *glvR* is involved in biofilm formation of *E. piscicida*, biofilm formation by the WT and Δ*glvR* strains was measured by crystal violet staining. The results showed that *glvR* deletion had no significant effect on biofilm formation (**Figure 6C**).

**Figure 6:**
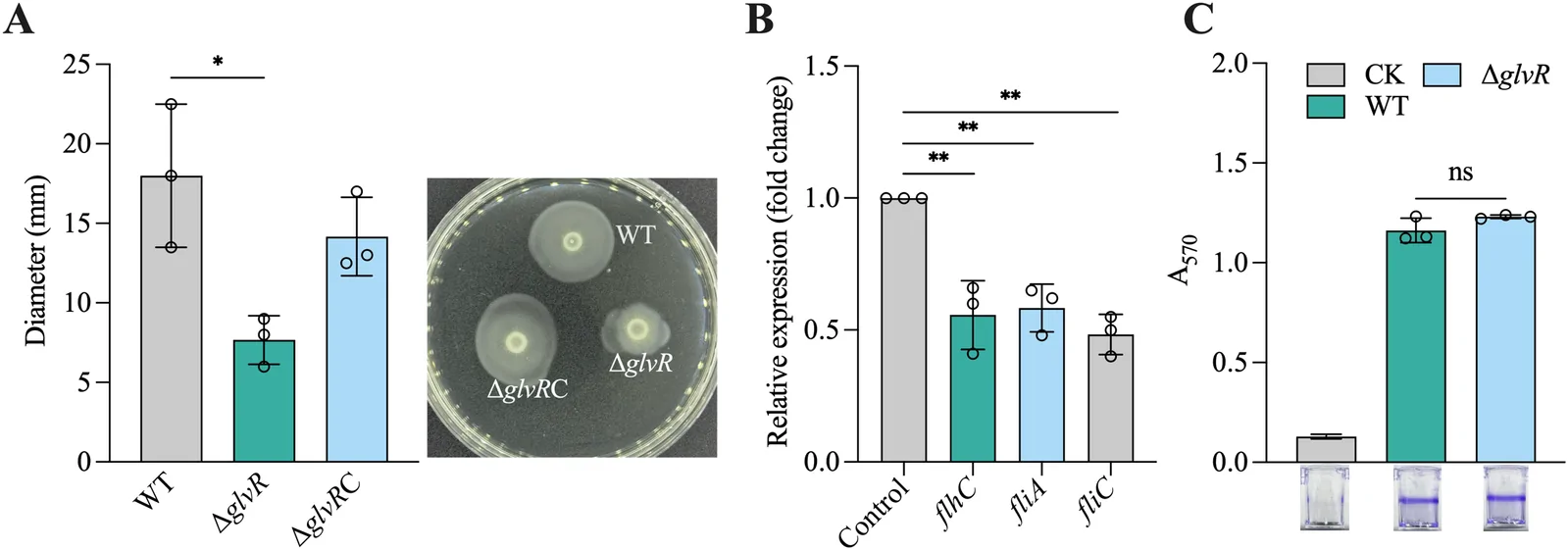
Effects of *glvR* on the motility and biofilm formation of *E. piscicida*. **(A)** Detection of bacterial motility on 0.3% semi-solid agar plates. Left panel: quantitative statistics of the motility ring diameters. **(B)** Measurement of the transcription levels of flagellum-associated genes. **(C)** Assessment of bacterial biofilm formation by crystal violet staining. Data are presented as the mean ± SEM of three independent experiments; two-group comparisons **(A, C)** were analyzed using the independent-samples t-test, and multi-group comparisons **(B)** were analyzed using one-way ANOVA followed by the Tukey–Kramer test,; *P < 0.05, **P < 0.01.

### 3.6 Effect of GlvR on the pathogenicity of *E. piscicida*

The aforementioned studies demonstrated that GlvR promotes glycerol utilization in *E. piscicida*. Notably, a well-defined functional coupling between metabolism and virulence has been established in *E. piscicida*. Previous studies have shown that this bacterium can sense host-derived mannose and form mannose-6-phosphate, thereby activating the transcriptional regulator EvrA, which in turn upregulates the key virulence regulator *esrB* and enhances T3SS/T6SS expression (Wei et al., 2019). On this basis, we hypothesized that GlvR-mediated glycerol metabolic remodeling may further affect the pathogenic process of *E. piscicida*; therefore, its pathogenicity-related phenotypes were further analyzed. First, the intracellular survival and proliferation of Δ*glvR* in murine RAW 264.7 macrophages were examined. The results showed that, compared with the WT, the number of viable intracellular bacteria of Δ*glvR* in RAW 264.7 cells was significantly reduced (**Figure 7A**), which was consistent with observations by laser confocal microscopy (**Figure 7B**). Further analysis revealed that the adhesion capacity of Δ*glvR* to the tilapia skin epithelial cell line TSE-04 was significantly lower than that of the WT (**Figures 7C, D**). To evaluate the role of GlvR in resisting host humoral immunity, the WT, Δ*glvR*, and Δ*glvR*C strains were each incubated with tilapia serum for 1 h, and bacterial survival was determined. The results showed that the survival rate of Δ*glvR* was significantly lower than those of the WT and the complemented strain (**Figure 7E**), indicating that GlvR contributes to the resistance of *E. piscicida* to killing by host serum. To further evaluate the role of *glvR* during *in vivo* infection, tilapia were infected with the WT, Δ*glvR*, and Δ*glvR*C strains, respectively, and bacterial colonization in the spleen and head kidney was determined. The results showed that, compared with the WT, the bacterial loads of Δ*glvR* in the spleen and head kidney were significantly reduced at both time points examined (**Figure 7F**), indicating that *glvR* deletion significantly weakened the tissue colonization capacity of *E. piscicida* in the host. The mortality of tilapia after infection was further monitored, and the results showed that the survival rate of tilapia in the Δ*glvR*-infected group was significantly higher than that in the WT-infected group, with a survival rate of 57% in the Δ*glvR* group versus only 10% in the WT group (**Figure 7G**), indicating that *glvR* deletion significantly reduced the lethality of *E. piscicida* to tilapia. Taken together, these results demonstrated that *glvR* deletion significantly impaired the adhesion of *E. piscicida* to host cells and its intracellular survival within macrophages, while also reducing serum tolerance and *in vivo* tissue colonization, ultimately attenuating its pathogenicity.

**Figure 7:**
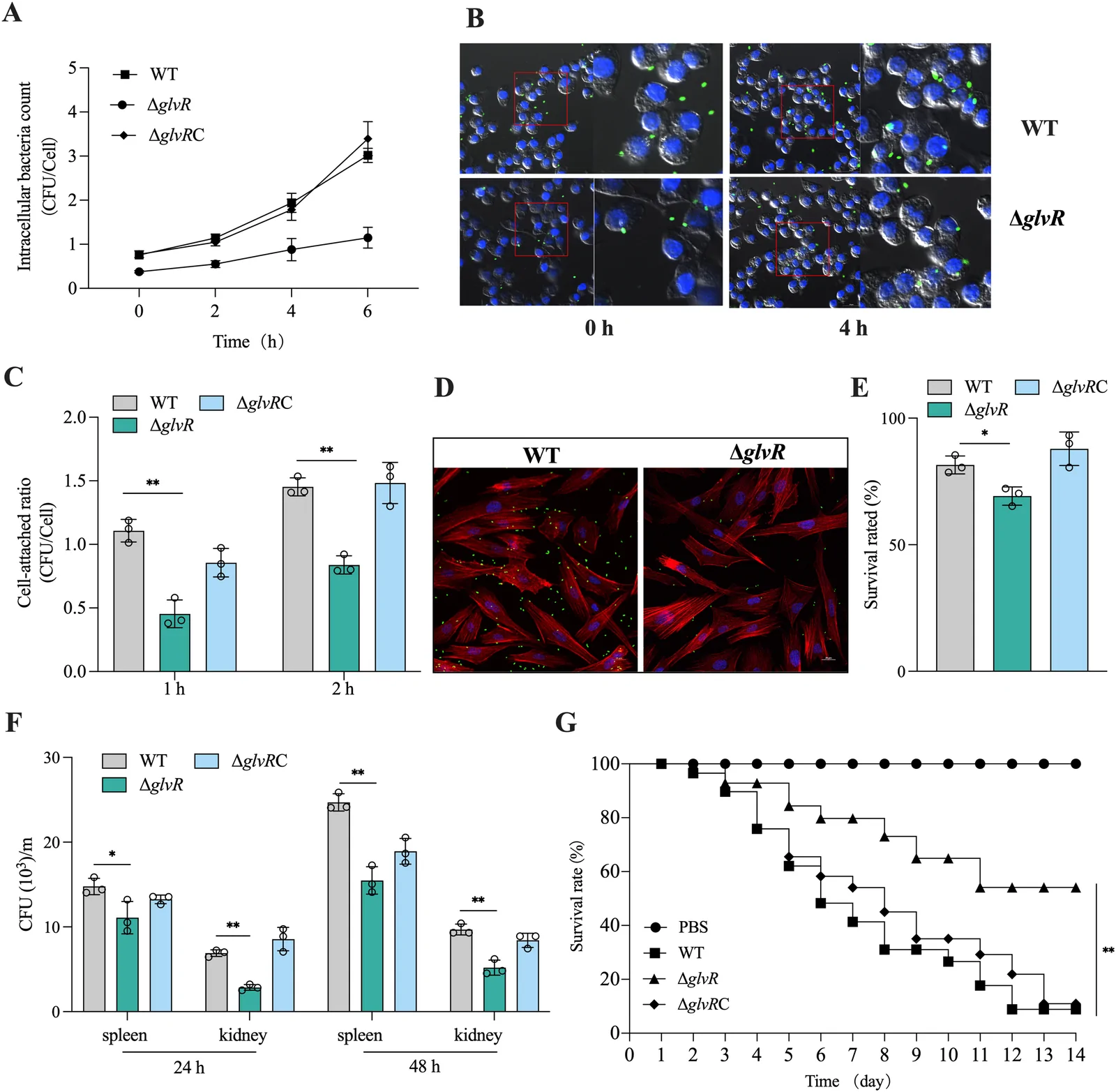
Effects of *glvR* deletion on the pathogenicity of *E. piscicida*. **(A)** Survival and replication within macrophages. Equivalent doses of the WT, Δ*glvR*, and Δ*glvR*C strains were incubated with murine RAW 264.7 macrophages for different time periods, and the intracellular bacterial load at each time point was determined by plate counting. **(B)** Observation of intracellular bacterial replication by confocal laser scanning microscopy (CLSM). Nuclei were stained blue with DAPI, and GFP-labeled bacteria are shown in green (scale bar: 20 μm). **(C)** Adhesion capacity to tilapia skin epithelial (TSE-04) cells. **(D)** Observation of *E. piscicida* adhesion to tilapia skin epithelial cells by CLSM. The cytoskeleton was stained red with phalloidin, nuclei were stained blue with DAPI, and GFP-labeled bacteria are shown in green (scale bar: 20 μm). **(E)** Resistance to killing by tilapia serum. **(F)** Assessment of the tissue colonization capacity in tilapia. **(G)** Survival rate of tilapia after challenge. Quantitative data are presented as the mean ± SEM of three independent experiments; two-group comparisons were analyzed using the independent-samples t-test,; differences in survival rates **(G)** were analyzed using the log-rank test; *P < 0.05, **P < 0.01.

### 3.7 CpxR directly and positively regulates the transcription of *glvR*

In other Gram-negative pathogens, regulatory links between the CpxRA system and AraC-like family regulators have been documented. For example, in *Shigella*, the expression of the AraC-family transcriptional regulator VirF is positively regulated by CpxR (Di Martino et al., 2016), whereas in *Yersinia*, the expression of LcrF, the master regulator of the Ysc–Yop type III secretion system, can be repressed by phosphorylated CpxR (Liu et al., 2012). These studies suggest that a regulatory coupling may exist between the Cpx stress response pathway and the AraC-family transcriptional regulatory network; however, this relationship has not been clarified in *E. piscicida*. To investigate whether *glvR* is regulated by the CpxRA system, the transcription levels of *glvR* in the Δ*cpxR*, Δ*cpxA*, and Δ*cpxRA* strains were first examined. The RT-qPCR results showed that *glvR* expression was significantly downregulated in all three mutants (**Figure 8A**). Furthermore, based on the reported canonical CpxR DNA-binding motif 5′-GTAAA-N5-GTAAA-3′, sequence analysis of the *glvR* promoter region identified a candidate binding sequence, 5′-GTATAAGTCTCTCTA-3′, which resembles this conserved motif, in the proximal promoter region (**Figure 8C**); we hypothesized this sequence to be a potential CpxR-binding site. Subsequently, the *glvR* promoter reporter vector pSglvR was co-transformed into DH5α with either pT3 (empty vector), pTcpxR (CpxR overexpression), or pTcpxRM (phosphorylation-deficient CpxR^D51A) for β-galactosidase activity assays. CpxR overexpression significantly increased *glvR* promoter activity compared with both the empty vector and CpxR^D51A (**Figure 8B**), indicating that CpxR positively regulates glvR and suggesting that this activation depends on CpxR phosphorylation. To further determine whether a direct interaction exists between CpxR and the *glvR* promoter, an electrophoretic mobility shift assay (EMSA) was performed using purified His6-CpxR protein. The results showed that His6-CpxR bound clearly to the *glvR* promoter probe, with migration shifts appearing as the protein concentration increased; upon addition of the unlabeled competitive probe, the binding signal was markedly weakened, whereas the control protein showed no binding (**Figure 8D**). These results demonstrated that CpxR can directly bind to the *glvR* promoter region and positively regulate the transcription of *glvR*.

**Figure 8:**
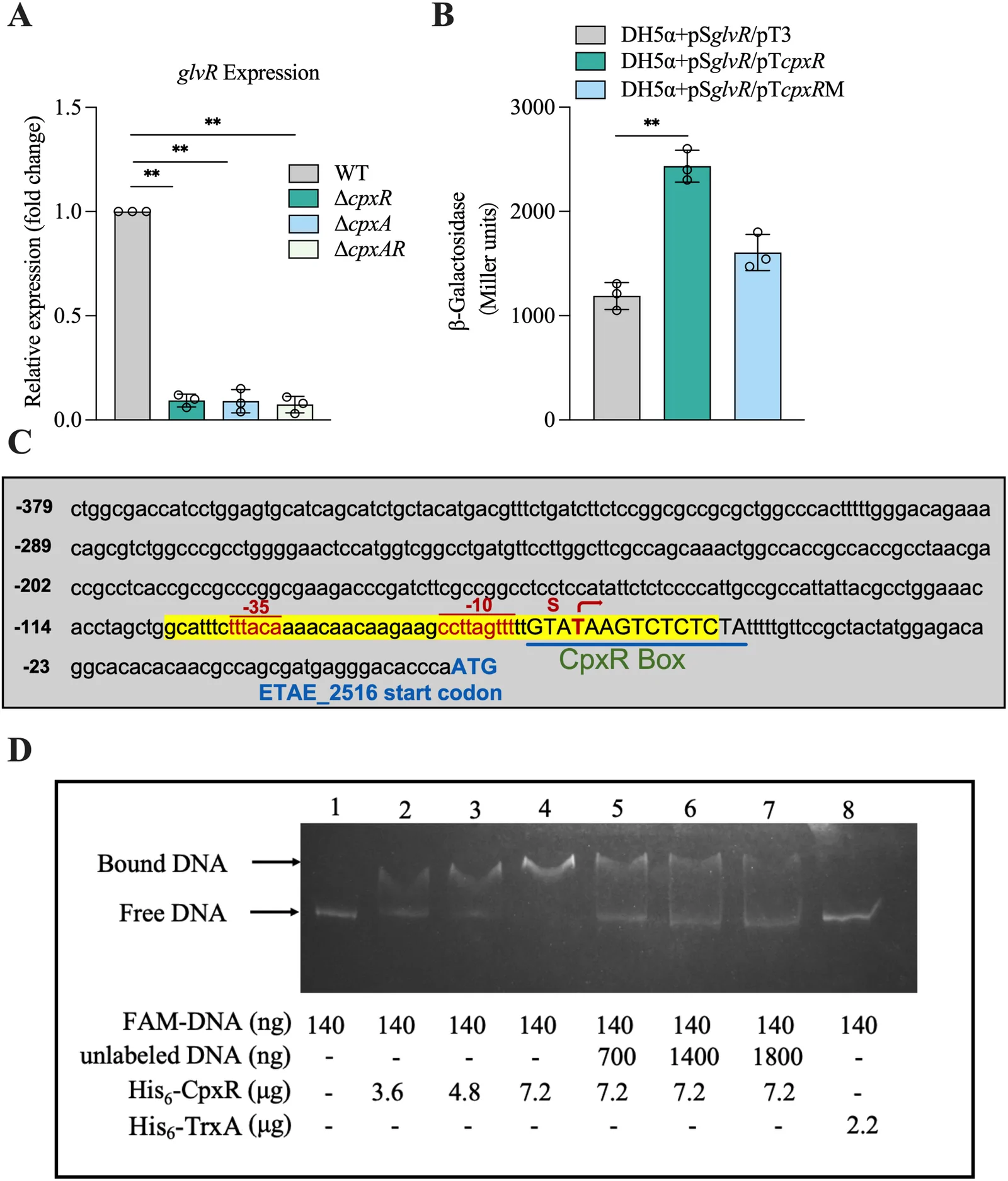
Transcription of *glvR* is positively regulated by CpxR. **(A)** Detection of *glvR* transcription levels by RT-qPCR. Transcription levels were measured in the wild-type strain and in the *cpxR*, *cpxA*, and *cpxRA* deletion mutants. **(B)** β-Galactosidase activity assay. **(C)** Prediction and analysis of CpxR-binding motifs in the *glvR* promoter. The predicted candidate CpxR-binding motif is marked with a blue underline, and the red letters indicate the transcription start site of *glvR*. **(D)** Detection of DNA–protein interaction by EMSA. FAM-labeled *glvR* promoter fragments were incubated with CpxR protein, subjected to non-denaturing PAGE, and visualized using a gel imaging system. Quantitative data are presented as the mean ± SEM of three independent experiments and were analyzed using one-way ANOVA followed by the Tukey–Kramer test; *P < 0.05, **P < 0.01.

## 4 Discussion

*Edwardsiella piscicida* is an important intracellular pathogen whose infection process depends on the rapid sensing of, and adaptive responses to, changes in the host intracellular environment. This involves, in particular, metabolic reprogramming under fluctuating nutrient availability and host defense pressure, thereby promoting intracellular survival, tissue colonization, and *in vivo* proliferation (Lanza et al., 2024; Peng et al., 2025). The results of this study indicate that GlvR is not a constitutive regulator required for basal bacterial growth, but is more likely an AraC-family transcriptional regulator that is activated under specific environmental conditions. By participating in the regulation of glycerol utilization, oxidative stress adaptation, and multiple virulence-associated phenotypes, GlvR collectively influences host adaptation and the infection process of *E. piscicida*. Sequence alignment showed that the amino acid sequence of GlvR is highly conserved among *E. piscicida*, *Edwardsiella tarda*, and several related Enterobacteriaceae species. Protein structure analysis further revealed that its C terminus harbors a typical AraC-like DNA-binding domain with a conserved helix-turn-helix (HTH) motif. This domain generally mediates specific DNA recognition through insertion of a recognition helix into the major groove of DNA, whereas environmental signal sensing by AraC-family proteins and the resulting conformational regulation are usually associated with their regulatory domains (Cortés-Avalos et al., 2021; Rodgers and Schleif, 2009). Taken together, these results suggest that GlvR may function as a conserved transcriptional regulator that links upstream stress sensing to downstream phenotypic outputs and participates in the adaptation of *E. piscicida* to environmental changes.

Our results further indicate that the core function of GlvR is primarily associated with glycerol utilization. Glycerol significantly induced *glvR* expression, whereas deletion of *glvR* downregulated the expression of multiple glycerol metabolism-related genes; moreover, GlvR positively enhanced the promoter activities of *glpA*, *glpD*, and *glpT*. These findings indicate that GlvR does not act as a broadly acting global regulator in *E. piscicida*, but rather as a condition-dependent regulatory node involved in the utilization of a specific carbon source. The principal feature of carbon catabolite repression (CCR) is the repression of genes involved in the utilization of non-preferred carbon sources in the presence of preferred carbon sources such as glucose, thereby establishing a hierarchy of carbon source utilization. AraC/XylS-family regulators frequently play important roles in this process by participating in the activation or remodeling of specific carbon metabolic pathways (Deutscher, 2008; Stülke and Hillen, 1999). Therefore, taken together with our results, GlvR more likely belongs to a class of transcriptional regulators that are activated under specific nutritional backgrounds and regulate glycerol uptake and utilization.

It is noteworthy that the physiological significance of glycerol metabolism is not limited to providing carbon and energy. Glycerol-3-phosphate (G3P) not only connects to central carbon metabolism but is also an important precursor of glycerophospholipid synthesis in eubacteria, and perturbations in its homeostasis can further affect membrane lipid composition, carbon flux allocation, and oxidative stress adaptation (Blötz and Stülke, 2017; Liu et al., 2022). In this study, we found that the Δ*glvR* mutant exhibited marked sensitivity to H_2_O_2_-induced oxidative stress, mainly reflected by a substantial decrease in survival and a significant increase in intracellular reactive oxygen species (ROS) levels. Meanwhile, exogenous H_2_O_2_ stimulation significantly upregulated the expression of multiple glycerol metabolism-related genes. These results indicate that GlvR does not broadly participate in tolerance to diverse adverse conditions, but instead exerts a specific regulatory role in oxidative stress adaptation. Metabolic reprogramming is considered a key strategy by which bacteria withstand environmental stress (Hadadi et al., 2020). Previous studies have shown that, in *E. tarda*, redirection of metabolic flux toward pyruvate can markedly enhance bacterial oxidative adaptation, and that pyruvate, as an α-keto acid, can scavenge intracellular H_2_O_2_ through a non-enzymatic reaction (Wang and Sun, 2023). In *Borrelia burgdorferi*, GpsA/GlpD-mediated glycerol-3-phosphate (G3P) metabolism is regarded as an important node linking pathogen infectivity with redox homeostasis (Drecktrah et al., 2022). Accordingly, we speculate that deletion of *glvR* impairs glycerol uptake and subsequent catabolism, which may further disturb G3P-associated metabolic balance and membrane lipid homeostasis, ultimately leading to increased ROS accumulation and reduced oxidative stress tolerance (Dowhan and Bogdanov, 2021; Liu et al., 2022). Therefore, the decreased oxidative stress tolerance caused by *glvR* deletion is most likely a secondary physiological consequence of impaired glycerol metabolism. This finding further clarifies how *E. piscicida* integrates carbon metabolism remodeling through GlvR to sustain survival under oxidative pressure in the complex host environment.

In addition to metabolism and stress resistance, GlvR further mediates flagellar motility and complex virulence-associated phenotypes of *E. piscicida*. Flagellum-driven motility is considered to be closely associated with the ability of various aquatic pathogens to breach host physical barriers, reach epithelial tissues, and accomplish early colonization (Manchanayake et al., 2023). In this study, the Δ*glvR* strain showed a significantly reduced motility diameter, and the transcription levels of *flhC*, the core factor of the flagellar regulatory cascade, and of its downstream effector genes *fliA* and *fliC* were significantly downregulated, suggesting that GlvR contributes to the maintenance of normal motility. In *E. tarda*, deletion of *flhC* has been demonstrated to abolish bacterial motility and to significantly weaken adhesion to and invasion of host cells (Xu et al., 2014). Consistently, we observed that the adhesion rate of Δ*glvR* to tilapia skin epithelial cells (TSE-04) was markedly decreased. Flagella are not merely motility organelles; in many pathogenic bacteria they also exert adhesive functions and can directly promote the initial contact between bacteria and host cells (Haiko and Westerlund-Wikström, 2013). Therefore, the reduced motility caused by *glvR* deletion is very likely one of the important reasons for its attenuated adhesion capacity. Further analyses showed that Δ*glvR* exhibited decreased intracellular survival and proliferation in RAW 264.7 cells, reduced survival in tilapia serum, and markedly lower bacterial loads in the spleen and head kidney, ultimately resulting in significantly increased survival of infected fish. These observations indicate that GlvR affects not a single isolated virulence factor, but multiple stages of the host infection process, including early adhesion, adaptation within phagocytes, resistance to serum immune clearance, and tissue colonization. In *E. piscicida*, a well-defined functional coupling between metabolism and virulence has been established. Previous studies have shown that this bacterium can sense host-derived mannose and convert it to mannose-6-phosphate, thereby activating the transcription factor EvrA, which upregulates *esrB* and enhances T3SS/T6SS expression; in addition, AdhE links sugar metabolism to virulence gene expression through its interaction with PtsH (Mao et al., 2022a; Wei et al., 2019). In summary, in *E. piscicida*, the metabolism of specific carbon sources is not only the physiological basis for acquiring nutrients and energy, but also serves as a key molecular cue for sensing the host microenvironment, thereby finely tuning the expression of its virulence program. The GlvR-mediated regulation of glycerol utilization revealed in this study provides new evidence supporting the coupling between metabolism and virulence in this bacterium.

AraC-family transcriptional regulators are widespread in bacteria and participate in diverse biological processes, including carbon source utilization, environmental stress responses, and the regulation of virulence factor expression (Gallegos et al., 1997; Tobes and Ramos, 2002). The CpxR/CpxRA two-component system is likewise an important regulatory module by which Gram-negative bacteria sense envelope and environmental stresses; its functions are generally not limited to primary stress responses, but can further influence metabolic adaptation and virulence-related processes through downstream regulators (Wan et al., 2024; Zhang et al., 2025). Therefore, elucidating how CpxR in *E. piscicida* transmits upstream environmental stress signals to metabolic adaptation and pathogenicity-related processes is an important prerequisite for understanding the hierarchical regulatory network between CpxR and AraC-like regulators. This study demonstrates that CpxR can directly bind to the *glvR* promoter region and positively activate its transcription, indicating a well-defined hierarchical regulatory relationship between CpxR and *glvR* in *E. piscicida*. Thus, GlvR does not function independently, but more likely acts as a specialized regulatory node downstream of CpxR, converting upstream stress sensing into glycerol metabolism remodeling and the associated virulence phenotypes. Notably, the CpxR-binding site of *glvR* identified in this study is not a classical consensus sequence but an atypical binding site, suggesting that CpxR recognition of different target genes may be differentiated to some extent, which would facilitate precise regulation under complex environmental conditions.

Several limitations of the present study should be acknowledged. First, although GlvR positively regulates glycerol metabolism and is simultaneously required for multiple virulence-associated phenotypes, the present data cannot fully distinguish whether the virulence attenuation of Δ*glvR* is directly caused by impaired glycerol utilization or by other GlvR-regulated processes, such as oxidative stress adaptation and flagellar motility; the designation “Glycerol-linked Virulence Regulator” is therefore intended to reflect the dual metabolic and virulence-associated functions of GlvR rather than a proven causal chain, and dissecting the causal contribution of each downstream branch will require further investigation. Second, the promoter reporter assays were performed only for *glpD*, *glpT*, and *glpA*; whether GlvR directly regulates the remaining downregulated genes, such as *glpF*, *glpK*, and *glpQ*, awaits further verification. Third, intracellular ROS levels were quantified in bacterial lysates using a microplate reader; flow cytometry-based analysis of individual live cells would provide higher resolution and will be considered in future work. Fourth, the direct interaction between CpxR and the *glvR* promoter was demonstrated *in vitro* by EMSA; *in vivo* evidence, such as chromatin immunoprecipitation (ChIP) assays, will further consolidate this conclusion. Finally, the direct effector genes through which GlvR regulates *flhC* expression and oxidative stress adaptation remain to be identified.

## 5 Conclusion

This study identifies GlvR as an AraC-family transcription factor that is directly and positively regulated by CpxR in *E. piscicida*. The available evidence indicates that GlvR more likely functions as a regulatory node activated under specific environmental conditions, participating in glycerol metabolism, oxidative stress adaptation, motility maintenance, and the regulation of virulence-associated phenotypes, and playing an important role in connecting envelope stress sensing, metabolic reprogramming, and pathogenic phenotypic output. The direct recognition by CpxR of an atypical binding site in the *glvR* promoter region further reveals the hierarchical and complex nature of the regulatory network downstream of this two-component system. This study provides new insights into the environmental adaptation and infection mechanisms of *E. piscicida* and lays a foundation for future dissection of its integrated metabolism–virulence regulatory network.

## Data Availability

The data presented in the study are deposited in the NCBI Sequence Read Archive (SRA) repository, BioProject accession number PRJNA1510749.

## References

[B1] Blötz C. Stülke J. (2017). Glycerol metabolism and its implication in virulence in Mycoplasma. FEMS Microbiol. Rev. 41, 640–652. doi: 10.1093/femsre/fux033 28961963

[B2] Cao L. Zhang Y. X. Choi S. Shao S. Wang Q. Y. (2025). The type III secretion system effector EseJ of *Edwardsiella piscicida* promotes intracellular colonization by regulating PPARγ expression in macrophages. J. Fish. China 49, 089416. doi: 10.11964/jfc.20250114863

[B3] Chakraborty S. Liu L. Fitzsimmons L. Porwollik S. Kim J.-S. Desai P. . (2020). Glycolytic reprograming in *Salmonella* counters NOX2-mediated dissipation of ΔpH. Nat. Commun. 11, 1783. doi: 10.1038/s41467-020-15604-2 32286292 PMC7156505

[B4] Cortés-Avalos D. Martínez-Pérez N. Ortiz-Moncada M. A. Juárez-González A. Baños-Vargas A. A. Estrada-de los Santos P. . (2021). An update of the unceasingly growing and diverse AraC/XylS family of transcriptional activators. FEMS Microbiol. Rev. 45, fuab020. doi: 10.1093/femsre/fuab020 33837749

[B5] Deutscher J. (2008). The mechanisms of carbon catabolite repression in bacteria. Curr. Opin. Microbiol. 11, 87–93. doi: 10.1016/j.mib.2008.02.007 18359269

[B6] Di Martino M. L. Falconi M. Micheli G. Colonna B. Prosseda G. (2016). The multifaceted activity of the VirF regulatory protein in the Shigella lifestyle. Front. Mol. Biosci. 3, 61. doi: 10.3389/fmolb.2016.00061 27747215 PMC5041530

[B7] Dowhan W. Bogdanov M. (2021). Eugene P. Kennedy’s legacy: defining bacterial phospholipid pathways and function. Front. Mol. Biosci. 8, 666203. doi: 10.3389/fmolb.2021.666203 33842554 PMC8027125

[B8] Drecktrah D. Hall L. S. Crouse B. Schwarz B. Richards C. Bohrnsen E. . (2022). The glycerol-3-phosphate dehydrogenases GpsA and GlpD constitute the oxidoreductive metabolic linchpin for Lyme disease spirochete host infectivity and persistence in the tick. PloS Pathog. 18, e1010385. doi: 10.1371/journal.ppat.1010385 35255112 PMC8929704

[B9] Eisenreich W. Heesemann J. Rudel T. Goebel W. (2013). Metabolic host responses to infection by intracellular bacterial pathogens. Front. Cell. Infect. Microbiol. 3, 24. doi: 10.3389/fcimb.2013.00024 23847769 PMC3705551

[B10] Fang Q. Wu Q. Huang H. Xu J. Huang J. Gu H. . (2023). TCS regulator CpxR of *Edwardsiella piscicida* is vital for envelope integrity by regulating the new target gene yccA, stress resistance, and virulence. Aquaculture 574, 739703. doi: 10.1016/j.aquaculture.2023.73970342574925

[B11] Gallegos M.-T. Schleif R. Bairoch A. Hofmann K. Ramos J. L. (1997). AraC/XylS family of transcriptional regulators. Microbiol. Mol. Biol. Rev. 61, 393–410. doi: 10.1128/.61.4.393-410.1997 PMC232617 9409145

[B12] Hadadi N. Pandey V. Chiappino-Pepe A. Morales M. Gallart-Ayala H. Mehl F. . (2020). Mechanistic insights into bacterial metabolic reprogramming from omics-integrated genome-scale models. NPJ Syst. Biol. Appl. 6, 1. doi: 10.1038/s41540-019-0121-4 32001719 PMC6946695

[B13] Haiko J. Westerlund-Wikström B. (2013). The role of the bacterial flagellum in adhesion and virulence. Biol. (Basel). 2, 1242–1267. doi: 10.3390/biology2041242 24833223 PMC4009794

[B14] Higgins D. E. Nazareno E. DiRita V. J. (1992). The virulence gene activator ToxT from *Vibrio cholerae* is a member of the AraC family of transcriptional activators. J. Bacteriol. 174, 6974–6980. doi: 10.1128/jb.174.21.6974-6980.1992 1400247 PMC207377

[B15] Hu Y.-H. Zhou H.-Z. Jin Q.-W. Zhang J. (2016). The serine protease autotransporter Tsh contributes to the virulence of *Edwardsiella tarda*. Vet. Microbiol. 189, 68–74. doi: 10.1016/j.vetmic.2016.04.021 27259829

[B16] Lanza A. Kimura S. Hirono I. Yoshitake K. Kinoshita S. Asakawa S. (2024). Transcriptome analysis of *Edwardsiella piscicida* during intracellular infection reveals excludons are involved with the activation of a mitochondrion-like energy generation program. mBio 15, e03526-23. doi: 10.1128/mbio.03526-23 38349189 PMC10936155

[B17] Lee W. VanderVen B. C. Fahey R. J. Russell D. G. (2013). Intracellular Mycobacterium tuberculosis exploits host-derived fatty acids to limit metabolic stress. J. Biol. Chem. 288, 6788–6800. doi: 10.1074/jbc.m112.445056 23306194 PMC3591590

[B18] Leung K. Y. Wang Q. Yang Z. Siame B. A. (2019). *Edwardsiella piscicida*: a versatile emerging pathogen of fish. Virulence 10, 555–567. doi: 10.1080/21505594.2019.1621648 31122125 PMC6592360

[B19] Lin C. Y. Jiang B. Zhang Y. M. Su Y. L. (2025). Research progress on *Edwardsiella piscicida* from fish. J. Fish. China 49, 029402. doi: 10.11964/jfc.20240914702

[B20] Liu J. Thanikkal E. J. Obi I. R. Francis M. S. (2012). Elevated CpxR∼P levels repress the Ysc–Yop type III secretion system of *Yersinia pseudotuberculosis*. Res. Microbiol. 163, 518–530. doi: 10.1016/j.resmic.2012.07.010 22842077

[B21] Liu Y. Sun W. Ma L. Xu R. Yang C. Xu P. . (2022). Metabolic mechanism and physiological role of glycerol 3-phosphate in *Pseudomonas aeruginosa* PAO1. mBio 13, e02624-22. doi: 10.1128/mbio.02624-22 36218368 PMC9765544

[B22] Ma D. Gu H. Shi Y. Huang H. Sun D. Hu Y. (2021). *Edwardsiella piscicida* YefM-YoeB: a type II toxin-antitoxin system that is related to antibiotic resistance, biofilm formation, serum survival, and host infection. Front. Microbiol. 12, 646299. doi: 10.3389/fmicb.2021.646299 33732226 PMC7957083

[B23] Manchanayake T. Salleh A. Amal M. N. A. Yasin I. S. M. Zamri-Saad M. (2023). Pathology and pathogenesis of *Vibrio* infection in fish: A review. Aquac. Rep. 28, 101459. doi: 10.1016/j.aqrep.2022.10145942574925

[B24] Mao Q. Jiang J. Wu X. Ma Y. Zhang Y. Zhao Y. . (2022a). Bifunctional alcohol/aldehyde dehydrogenase AdhE controls phospho-transferase system sugar utilization and virulence gene expression by interacting PtsH in *Edwardsiella piscicida*. Microbiol. Res. 260, 127018. doi: 10.1016/j.micres.2022.127018 35405475

[B25] Mao Q. Jiang J. Wu X. Xu R. Ma Y. Zhang Y. . (2022b). Coordinate regulation of carbohydrate metabolism and virulence by PtsH in pathogen *Edwardsiella piscicida*. Appl. Microbiol. Biotechnol. 106, 2063–2077. doi: 10.1007/s00253-022-11848-8 35218391 PMC8881556

[B26] Nakayama S.-I. Watanabe H. (1998). Identification of *cpxR* as a positive regulator essential for expression of the *Shigella sonnei virF* gene. J. Bacteriol. 180, 3522–3528. doi: 10.1128/jb.180.14.3522-3528.1998 9657992 PMC107317

[B27] Parsons J. B. Rock C. O. (2013). Bacterial lipids: metabolism and membrane homeostasis. Prog. Lipid Res. 52, 249–276. doi: 10.1016/j.plipres.2013.02.002 23500459 PMC3665635

[B28] Peng Y. Liu Y. Wu J. Zhang Y. Wang Q. Shao S. (2025). ArgR-dependent bacterial resistance to host lipid droplets in *Edwardsiella piscicida*. Commun. Biol. 8, 333. doi: 10.1038/s42003-025-07777-7 40021749 PMC11871307

[B29] Qin L. Wang X. Gao Y. Bi K. Wang W. (2020). Roles of EvpP in *Edwardsiella piscicida*-macrophage interactions. Front. Cell. Infect. Microbiol. 10, 53. doi: 10.3389/fcimb.2020.00053 32117819 PMC7033576

[B30] Rodgers M. E. Schleif R. (2009). Solution structure of the DNA binding domain of AraC protein. Proteins 77, 202–208. doi: 10.1002/prot.22431 19422057 PMC2745637

[B31] Shi Y.-J. Fang Q.-J. Huang H.-Q. Gong C.-G. Hu Y.-H. (2019). HutZ is required for biofilm formation and contributes to the pathogenicity of *Edwardsiella piscicida*. Vet. Res. 50, 76. doi: 10.1186/s13567-019-0693-4 31578154 PMC6775658

[B32] Stülke J. Hillen W. (1999). Carbon catabolite repression in bacteria. Curr. Opin. Microbiol. 2, 195–201. doi: 10.1016/s1369-5274(99)80034-4 10322165

[B33] Tobes R. Ramos J. L. (2002). AraC-XylS database: a family of positive transcriptional regulators in bacteria. Nucleic Acids Res. 30, 318–321. doi: 10.1093/nar/30.1.318 11752325 PMC99111

[B34] Veleba M. Higgins P. G. Gonzalez G. Seifert H. Schneiders T. (2012). Characterization of RarA, a novel AraC family multidrug resistance regulator in *Klebsiella pneumoniae*. Antimicrob. Agents Chemother. 56, 4450–4458. doi: 10.1128/aac.00456-12 22644028 PMC3421627

[B35] Wan J. Gao X. Liu F. (2024). Regulatory role of the Cpx ESR in bacterial behaviours. Virulence 15, 2404951. doi: 10.1080/21505594.2024.2404951 39292643 PMC11790278

[B36] Wang B.-Y. Huang H.-Q. Li S. Tang P. Dai H.-F. Xian J.-A. . (2019). Thioredoxin H (TrxH) contributes to adversity adaptation and pathogenicity of *Edwardsiella piscicida*. Vet. Res. 50, 26. doi: 10.1186/s13567-019-0645-z 30992061 PMC6466703

[B37] Wang Q. Yang M. Xiao J. Wu H. Wang X. Lv Y. . (2009). Genome sequence of the versatile fish pathogen *Edwardsiella tarda* provides insights into its adaptation to broad host ranges and intracellular niches. PloS One 4, e7646. doi: 10.1371/journal.pone.0007646 19865481 PMC2764856

[B38] Wang X. Sun B. (2023). Metabolic proteins with crucial roles in *Edwardsiella tarda* antioxidative adaptation and intracellular proliferation. mSystems 8, e00391-23. doi: 10.1128/msystems.00391-23 37729581 PMC10654080

[B39] Wei L. Qiao H. Sit B. Yin K. Yang G. Ma R. . (2019). A bacterial pathogen senses host mannose to coordinate virulence. iScience 20, 310–323. doi: 10.1016/j.isci.2019.09.028 31605945 PMC6817725

[B40] Westman J. Grinstein S. (2021). Determinants of phagosomal pH during host-pathogen interactions. Front. Cell Dev. Biol. 8, 624958. doi: 10.3389/fcell.2020.624958 33505976 PMC7829662

[B41] Xu T. Su Y. Xu Y. He Y. Wang B. Dong X. . (2014). Mutations of flagellar genes *fliC12*, *fliA* and *flhDC* of *Edwardsiella tarda* attenuated bacterial motility, biofilm formation and virulence to fish. J. Appl. Microbiol. 116, 236–244. doi: 10.1111/jam.12357 24118854

[B42] Yang J. Tauschek M. Robins-Browne R. M. (2011). Control of bacterial virulence by AraC-like regulators that respond to chemical signals. Trends Microbiol. 19, 128–135. doi: 10.1016/j.tim.2010.12.001 21215638

[B43] Zhang M. Sun K. Sun L. (2008). Regulation of autoinducer 2 production and *luxS* expression in a pathogenic *Edwardsiella tarda* strain. Microbiology 154, 2060–2069. doi: 10.1099/mic.0.2008/017343-0 18599834

[B44] Zhang S. Y. Sun S. S. Liu L. Y. Sivaranjan T. Nie P. Xie H. X. (2025). The two-component system CpxAR controls biofilm formation by directly regulating the T3SS needle tip protein EseB in *Edwardsiella piscicida*. Appl. Environ. Microbiol. 91, e02264-24. doi: 10.1128/aem.02264-24 40439422 PMC12175530

