# Supplementary material for: The CpxR–GlvR regulatory axis of Edwardsiella piscicida: a novel virulence regulatory mechanism integrating glycerol metabolism and oxidative stress adaptation
Source: Front Cell Infect Microbiol. 2026 Sep 25;16:1963223. doi: 10.3389/fcimb.2026.1963223 (PMC13651467; doi:10.3389/fcimb.2026.1963223)
Supplement: Supplementary file 1 [file Table1.docx]

# Tables

**Supplementary Table 1** Strains, plasmids, and cell lines used in this study.

| Strain or plasmid | Description/characteristics | Source/culture conditions |
| --- | --- | --- |
| *Edwardsiella piscicida* TH01 | Wild-type strain | Institute of Oceanology, Chinese Academy of Sciences; LB medium, 28°C |
| *Escherichia coli* S17-1λpir | Conjugative donor strain for plasmid transfer | Institute of Oceanology, Chinese Academy of Sciences; LB medium, 37°C |
| Trans1-T1 | Chemically competent *E. coli* cells for cloning | TransGen Biotech (Beijing, China); LB medium, 37°C |
| pDM4 | Suicide plasmid; Cm^R | Institute of Oceanology, Chinese Academy of Sciences; chloramphenicol |
| pT3 | Cloning/complementation vector; Amp^R | Institute of Oceanology, Chinese Academy of Sciences; ampicillin |
| pSC11 | Promoter-probe (reporter) vector; Km^R | Institute of Oceanology, Chinese Academy of Sciences; kanamycin sulfate |
| TSE-04 | Tilapia skin epithelial cell line | College of Fisheries, Guangdong Ocean University; L-15 medium |
| RAW 264.7 | Murine macrophage cell line | Institute of Oceanology, Chinese Academy of Sciences; DMEM medium |

**Supplementary Table 2** Primers used in this study

| Primers name | Sequence (5′→3′) | Description |
| --- | --- | --- |
| GlvKOF1 | GGTTACCCGCATGCAagatctTGCATC  AGCATCTGCTACATGA (*BglII*) | Construction of *glvR* deletion mutant strain |
| GlvKOR1 | GGTTACCCGCATGCAagatctCCCGGC  AGCTATCGCCAG |  |
| GlvKOF2 | GGGGCCCTTCTAGATagatctAATAC  GCTTGTCCAGATTCTGCT |  |
| GlvKOR2 | GGGGCCCTTCTAGATagatctCGGGTTGA  GCTGTTGATCGG (*BglII*) |  |
| GlvCF | ATCCAAGGAGATATAcatatgATG  ACAACCCAGGGACAGCA (*NdeI*) | Complementation strain construction |
| GlvCR | gtggtggtggtggtgctcgagG  GCGAGCGATAGCGCAAC (*XhoI*) |  |
| ET test F2 | GGAATTTCACTATGACCGTGAC | Detection primers for *E. piscicida* |
| ET test R2 | GCGAGAACACCGCCTTCT |  |
| BRF4 | GAAAAGTGCCACCTGACGT | Detection primers for pT3 plasmid |
| rrnB-R2 | CGGCGTTTCACTTCTGAGTT |  |
| *glpA*-RTF  *glpA*-RTR  *glpD*-RTF  *glpD*-RTR  *glpT*-RTF  *glpT*-RTR  *glpK*-RTF  *glpK*-RTR  *glpF*-RTF  *glpF*-RTR  *glpQ*-RTF  *glpQ*-RTR  *glpR*-RTF | ATCATGAGGTGTGCGGACTG  ACCACGATCTGTGCGTGAAT  CCGCTATGCCCATACCTACG  GTGTTCGCCTAAGTCAGCCA  CTCGCGATGCCTTATCTGGT  AGACGGAGCCCATGATGAAC  CCTCGACCACGATGCCAATA  TGAGGGTAGAGCTCTGGGTC  CAGATCTCCATCGGGTCGTG  GATCGCCGTATCCATCAGCA  TGATTTACCCGACCAACCCG  GGCCTGCGCTTCAAAATAGG  CGCGATGGTAAATCTGGGGA | RT-qPCR |
| *glpR*-RTR  *flhC-*RTF  *flhC-*RTR | GATCTCCAGCTGAACGCTGT  CGGGGTAGAGGCGGTGAT  CGCATGGGCAATAAAGCT |  |
| *fliA*-RTF | GCCGATCTCCTTGAGGTTGA |  |
| *fliA*-RTR | ACGACGAATGGCGTGAGG |  |
| *fliC-*RTF | CTTTCTTGATAGAGGTTACGGCTG |  |
| *fliC-*RTR | GATGCGACATTAAAAGCTGACTC |  |
| GlvR*-*PF | CGCTGCACCAGTTTCGGATCCTG  CATCAGCATCTGCTACATGA | GlvR promoter |
| GlvR-PR | AATTTAAATCCCATCGGATC  CTGGGTGTCCCTCATCGCTG |  |
| GlpA-PF | CGCTGCACCAGTTTCGGATCCTG  ATCAAAATCACTCTACAGACACGC | GlpA promoter |
| GlpA-PR | AATTTAAATCCCATCGGATCCAAA  TATCTCCGATGACTGTTATTGTTTG |  |
| GlpD-PF | CGCTGCACCAGTTTCGGATCC  GACTCCTCCCGCTCTCTGCA | GlpD promoter |
| GlpD-PR | AATTTAAATCCCATCGGATCCG  ATACCTCGTCACGGCCTATGG |  |
| GlpT-PF | CGCTGCACCAGTTTCGGATCC  ATTTGAACGTTGCGAAGCAAC | GlpT promoter |
| GlpT-PR | AATTTAAATCCCATCGGATCCCT  ATGCGGCATCAGAGCAGAT |  |
| pT*glvR*-F | ATCCAAGGAGATATACATATGAT  GACAACCCAGGGACAGCA | *glvR* overexpression |
| pT*glvR*-R | GTGGTGGTGGTGGTGCTCGA  GGGCGAGCGATAGCGCAAC |  |

Note: The nucleotides underlined are the restriction enzyme cleavage sites
